# Effects of Supplementation with Bee Pollen and Propolis on Growth Performance and Serum Metabolites of Rabbits: A Meta-Analysis

**DOI:** 10.3390/ani13030439

**Published:** 2023-01-27

**Authors:** María Inés Sierra-Galicia, Raymundo Rodríguez-de Lara, José Felipe Orzuna-Orzuna, Alejandro Lara-Bueno, Rodolfo Ramírez-Valverde, Marianela Fallas-López

**Affiliations:** 1 Posgrado en Producción Animal, Departamento de Zootecnia, Universidad Autónoma Chapingo, Texcoco, CP 56230, Mexico; 2 “Conejos” Centro de Investigación Científica del Estado de México A.C. (COCICEMAC), Coatlinchán, CP 56250, Mexico

**Keywords:** growth promoters, serum metabolites, honeybee products, antioxidant status

## Abstract

**Simple Summary:**

Bee pollen and propolis have been used successfully to improve performance and serum metabolites in poultry; however, their effects in rabbits have been inconsistent. Therefore, the objective of this study was to evaluate the supplementation with bee pollen and propolis on animal performance and serum metabolites of rabbits through a meta-analysis. In rabbits, supplementation with bee pollen and propolis has been shown to reduce the feed conversion rate; however, it can also increase weight gain and total antioxidant capacity in blood serum. These results suggest that bee pollen and propolis could be used as natural growth promoters and to improve rabbits’ antioxidant status.

**Abstract:**

The objective of this study was to evaluate the effects of bee pollen (BP) and propolis (PRO) supplementation on rabbits’ productive performance and serum metabolites through a meta-analysis. Sixteen peer-reviewed publications were included in the data set. The rabbit strains used in the studies included in the data set were New Zealand White, V-line, Rex, and V-line crosses. Weighted mean differences (WMD) between treatments supplemented with BP or PRO and control treatments were used to assess the magnitude of the effect. BP supplementation decreased (*p* < 0.001) daily feed intake (DFI) and feed conversion ratio (FCR); however, increased (*p* < 0.001) average daily gain (ADG) and hot carcass yield (HCY). PRO supplementation reduced DFI (*p* = 0.041) and FCR (*p* < 0.001), and increased ADG (*p* < 0.001) and HCY (*p* = 0.005). In blood serum, BP supplementation increased total antioxidant capacity (TAC; *p* = 0.002) and decreased serum creatinine concentration (*p* = 0.049). Likewise, decreased serum levels of aspartate aminotransferase (AST), alanine aminotransferase (ALT), and malondialdehyde (MDA) were detected in response to BP supplementation (*p* < 0.05). PRO supplementation increased the TAC in blood serum (*p* = 0.018); however, decreased serum concentrations of AST, ALT, and MDA were observed (*p* < 0.05). In conclusion, BP or PRO supplementation can be used as a natural growth promoter in rabbits, and both can also improve rabbits' antioxidant status. However, BP or PRO supplementation does not affect rabbits' renal or hepatic health status.

## 1. Introduction

It is necessary to increase the number and productivity of weaned rabbits and reduce mortality during the growth period to improve profitability in rabbit farms [1]. Therefore, in diets for growing rabbits, it is common to include antibiotics (for example, zinc bacitracin) that reduce the incidence of diseases and act as growth promoters [2]. However, the indiscriminate use of antibiotics contributes to the increase in the appearance of bacteria resistant to their effects, representing a significant threat to the health of animals and humans [3]. Consequently, the use of antibiotics as growth promoters has been prohibited in several countries, representing a major challenge for rabbit meat producers [4]. For these reasons, in recent years, the interest of researchers in the search and development of new natural alternatives to antibiotics and synthetic antioxidants has increased [4]. Among the natural alternatives currently available are products derived from bees (*Apis mellifera*), such as bee pollen (BP) and propolis (PRO). These products contain various bioactive metabolites with pharmaceutical properties [5].

BP is a mixture of nectar, salivary secretions from bees, and pollen grains collected from flowers [6]. According to Martinello and Mutinello [7], BP is composed of proteins (5–60%), sugars (13–55%), crude fiber (0.3–20%), lipids (4–7%), minerals, and phenolic compounds, mainly flavonoids (3–8%). BP has been reported to have various therapeutic properties, including antioxidant, anti-inflammatory, immunomodulatory, and antimicrobial activity [6]. On the other hand, PRO is a resinous substance that bees produce by mixing salivary gland secretions with beeswax and plant exudates [5]. PRO is mainly composed of flavonoids and phenolic acids (40–70%), waxes (20–35%), essential oils (1–3%), and approximately 5% of other organic substances [8]. In addition, PRO has been reported to have antioxidant, anti-inflammatory, antifungal, and antimicrobial properties [5]. In animal science, the effects of BP and PRO supplementation have been evaluated primarily in poultry [9,10]. However, the information available on the effects of BP and PRO supplementation in rabbits is still limited. In several species of domestic animals (for example, sheep and broilers, among others), it has been reported that supplementation with BP or PRO improves the immune response [11], increases feed digestibility [12], reduces oxidative stress [13], and improves animal performance [9].

Particularly in rabbits, studies have been conducted to evaluate the effects of BP and PRO supplementation on productive performance [2,14], carcass yield [15,16], antioxidant status in blood [1,17], and blood biochemistry [18,19]. In addition, some studies have reported that BP or PRO supplementation can effectively replace zinc bacitracin (the main antibiotic used in rabbits) in rabbit diets without affecting performance, mortality, or economic profitability. [4,20]. However, the results obtained to date have yet to be homogeneous or conclusive. The variability in doses, experimental periods, supplementation methods, and age of the animals are associated with the heterogeneity of the results observed in rabbits supplemented with BP and PRO [19]. These sources of variability must be identified and controlled to develop products containing BP and PRO that can be used as food supplements to improve rabbits’ productive performance and health.

A few review articles have been published [5,6,10], mentioning that BP or PRO supplementation can be used to improve productive and reproductive performance and the health of mammals and poultry. However, none of these review articles focused only on rabbits, nor did they use meta-analytic methods. Meta-analysis (MA) is a statistical tool that estimates the average effect of a given intervention through the combination and quantitative synthesis of results previously published in different studies [21]. Additionally, the MA makes it possible to identify sources of heterogeneity between studies [22]. Although the use of MA in research related to animal nutrition is proliferating, in rabbit nutrition, the use of MA is still limited [23]. The present study hypothesizes that supplementation with BP or PRO will benefit the productive performance of rabbits without affecting their health. Therefore, the objective of this meta-analysis was to evaluate the effects of BP and PRO supplementation on animal performance, carcass yield, oxidative status, and serum metabolites of rabbits.

## 2. Materials and Methods

### 2.1. Literature Search and Study Selection

**Figure A1:**
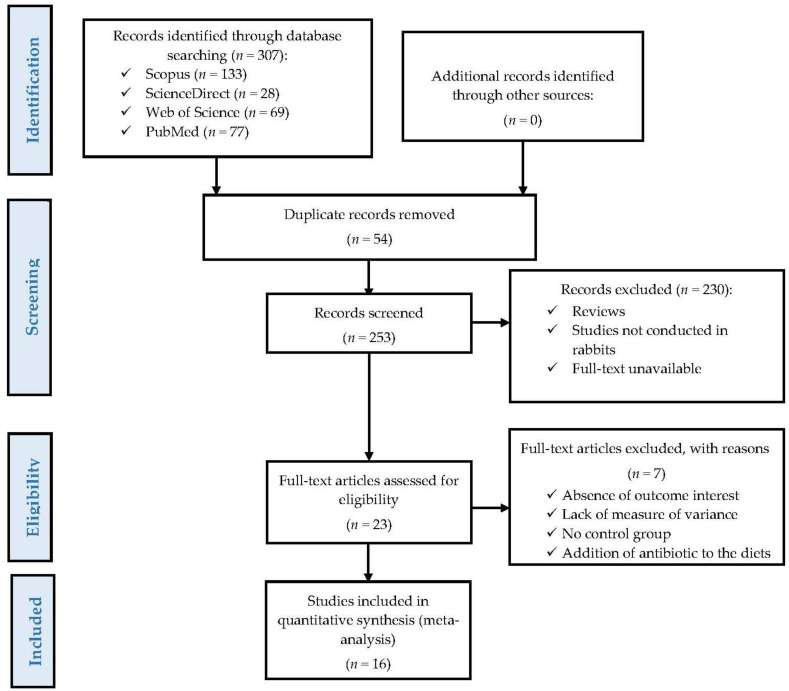
A PRISMA flow diagram detailing the literature search strategy and study selection for the meta-analysis.

A meta-analysis was performed to assess the effects of BP and PRO supplementation on rabbits’ productive performance and serum metabolites. For this, an exhaustive and structured search of scientific articles focused on evaluating the effects of supplementation with BP or PRO was carried out, following the PRISMA guidelines [24] in the identification, selection, choice, and inclusion of studies (Figure A1). The Scopus, PubMed, Web of Science, and ScienceDirect databases were used for the search process. The keywords used in the four databases were the following: rabbit, bee pollen, propolis, growth performance, carcass yield, and blood metabolites. Search results were restricted to studies published between January 2000 and November 2022, and 307 scientific publications were identified (Figure A1). Duplicate publications were excluded from the database. The remaining publications were subjected to a two-step selection process, as previously reported by other authors [25,26,27]. 

For this process, the titles and abstracts of each publication were first reviewed. Based on this information, all studies that were not conducted in rabbits, those that used experimentally infected rabbits, those that did not measure any of the variables of interest, and review articles were excluded. In the second step of the selection process, the articles analyzed had to meet some inclusion criteria to be considered in the final database. The inclusion criteria applied in the present meta-analysis were similar to those previously reported by other authors [23,26,28]: (1) studies using rabbits housed in cages (total confinement); (2) data on productive performance or serum metabolites; (3) studies using control and experimental treatments fed similarly, except for BP or PRO supplementation; (4) studies that reported the doses of BP or PRO used, or that contained sufficient information to estimate the doses of BP or PRO given to rabbits; (5) studies published in peer-reviewed scientific journals and written in English; and (6) studies reporting the treatment means (control and experimental), the standard error or standard deviation, and the number of replicates.

### 2.2. Data Extraction

Considering the inclusion criteria previously described in the database used for the meta-analysis, only 16 articles were included (Table A1). Furthermore, of the articles included in the final database, only data for response variables reported in at least three studies were extracted [25,27,28]. Consequently, in the present meta-analysis, variables of animal performance (weight gain, daily feed intake, and feed conversion rate) and hot carcass yield were included. In addition, the serum concentration of urea, creatinine, cholesterol, albumin, globulin, total protein, liver enzymes (aspartate aminotransferase and alanine aminotransferase), malondialdehyde, and total antioxidant capacity in blood serum were included. 

For each of the variables mentioned, the means of the control (without supplementation) and experimental treatments (supplemented with BP or PRO), the standard deviations (SD), and the number of repetitions (n) were extracted. When an article did not report the SD, it was calculated using the following equation [29]: SD = SEM × √n, where SEM = standard error of the treatment means. Additionally, from each of the selected publications (n = 16), the following complementary information was obtained: (1) author and year of publication, (2) country where the study was conducted, (3) nutritional composition of the experimental diets (g/kg DM), (4) duration of BP or PRO supplementation period (days), (5) dose of BP or PRO used (mg/kg BW), (6) age of rabbits, and (7) sex and rabbit strain. 

### 2.3. Calculations and Statistical Analysis

Statistical analyses of meta-analysis, analysis of heterogeneity, publication bias, meta-regression, and subgroup analysis were performed using the 'metafor' [30] package of R statistical software version 4.1.2 (R Core Team, Vienna, Austria). The effects of BP or PRO supplementation in rabbits were determined using the weighted mean differences (WMD) between the experimental treatments (rabbits supplemented with BP or PRO) and control treatments (rabbits not supplemented with BP or PRO). In the present study, the WMD was used because it allows the interpretation of the results obtained in the original units of measurement [31]. The treatment means for all the evaluated variables were weighted by the inverse of the variance, according to the method previously proposed by Der-Simonian and Laird [32] for random effects models.

Descriptive statistical values were obtained for the nutritional composition of the diets used using the PROC MEANS procedure of the SAS statistical software [33]. The SAS PROC MIXED procedure was used to determine the differences in the nutritional composition of the diets used in the treatments supplemented with BP or PRO and the control treatments. For this, the different studies were included as a random effect, and the Tukey test was used to detect possible statistical differences (*p* ≤ 0.05) between the treatments, as previously described by other authors [26,27].

### 2.4. Heterogeneity and Publication Bias

In the present meta-analysis, the heterogeneity of the effect of the treatments (variability between studies) was determined using the statistical tests of chi-square (Q) and I^2^ (percentage of variation) [22]. For the Q test, a significance level of *p* ≤ 0.10 was used, as its power has been reported to be relatively low in detecting heterogeneity among a small number of comparisons [34]. On the other hand, the I^2^ statistical test was used to measure heterogeneity as a percentage [35]. In the I^2^ test, the values are between 0 and 100%; values less than 25, between 25 and 50, and greater than 50% indicate low, moderate, and high heterogeneity, respectively [21,22].

Egger’s linear regression asymmetry test was used to assess the presence of publication bias [36]. This test was considered statistically significant when *p* ≤ 0.05 was obtained. Additionally, when a significant bias was detected (*p* ≤ 0.05) with Egger's test, the "trim and fill" method of Duval and Tweedie [37] was applied to determine the number of missing observations.

### 2.5. Meta-regression and Subgroup Analysis

Meta-regression analyses were performed to investigate potential sources of heterogeneity in the response variables tested. The variables had to meet the following meta-regression criteria: (1) variables reported in at least ten different studies [38]; (2) *p*-value ≤ 0.10 for the Q or I^2^ test greater than 50% [21,35]; and (3) *p*-value ≥ 0.05 for the Egger regression asymmetry test [37]. For the meta-regression, the methods of Der-Simonian and Laird [32] were followed since these procedures are well established to estimate the between-study variance. In cases where any covariate was significant with a *p*-value ≤ 0.05, a subgroup analysis was applied to the WMD. First, the supplementation method, the rabbits’ age, sex, and rabbit strain were used as categorical covariates. Next, the length of the experimental period (days) and doses (mg/kg BW) were used as continuous covariates. Subsequently, the statistically significant covariates (*p* ≤ 0.05) were evaluated by subgroup analysis [25,26,27]. The supplementation method covariate was divided into the following subgroups: (1) oral aqueous solution with a syringe, (2) capsules taken orally, and (3) orally through drinking water. The covariate rabbit sex was divided into three subgroups: (1) male rabbits, (2) female rabbits, and (3) mixed male and female rabbits (50% of each). The covariate rabbit strain was divided into four subgroups: (1) New Zealand White, (2) Rex, (3) V-line, and (4) V-line crosses. In addition, the covariate age of the rabbits was divided into two subgroups: (1) ≤ 15 weeks and (2) > 15 weeks. The continuous covariates that were significant in the meta-regression were evaluated using the following subgroups: supplementation period (≤ 70 and > 70 days) and dose used (≤ 350 and > 350 mg/kg BW). The reference values of the covariates were established based on the median values obtained with the descriptive statistical analysis performed on each covariate. For example, the age and the experimental period median were 15 weeks and 70 days, respectively. In the case of the dose, the median was 335 mg/kg BW, but we decided to close the amount to 350 mg/kg BW.

## 3. Results

### 3.1. Study Attributes and Excluded Studies

Table 1 shows no statistical differences (*p* > 0.05) between the control treatment and the one supplemented with BP for the nutrient content of the diet. Similarly, no differences (*p* > 0.05) were detected between the control treatment and the PRO-supplemented treatment for any of the dietary components (Table A2). These results suggest that, for our data set, it is possible to exclude the effects of dietary nutrients on the response of rabbits to BP or PRO supplementation.

The studies included in the present meta-analysis were conducted in only four countries. In summary, studies evaluating BP were conducted in Egypt (81.8%), Brazil (9.1%), and Mexico (9.1%). Similarly, studies evaluating PRO were conducted in Egypt (62.5%), Saudi Arabia (12.5%), Mexico (12.5%), and Brazil (12.5%). Table 1 shows that the doses of BP used varied between 100 and 1000 mg/kg BW. The doses of PRO used were between 30 and 846 mg/kg BW (Table A2). The experimental periods of the studies using BP ranged from 28 to 140 days (Table 1). Table A2 shows that the studies that evaluated PRO used experimental periods of 32 to 140 days. In most treatments that evaluated BP, the rabbits used were > 15 weeks old (70.3%) and only 29.7% of the treatments used rabbits that were ≤ 15 weeks old. The treatments that evaluated PRO mainly used rabbits that were ≤ 15 weeks of age (80.0%), and only 20.0% of the treatments used rabbits that were > 15 weeks of age. Regarding the supplementation method, most treatments (67.7%) supplied the BP in an aqueous solution using an oral syringe. Likewise, 25.9% of the treatments supplied the BP orally through drinking water, and the remaining treatments (7.4%) supplemented the BP in capsules. In the treatments that evaluated PRO, this product was supplemented mixed with the basal diet (66.7%) by oral aqueous solution with a syringe (20.0%) and using capsules (13.3%). Most studies (50%) used male rabbits, 18.7% used female rabbits, 18.7% used mixtures of male and female rabbits (50% of each), and 12.6% of the studies did not report the sex of the rabbits used. Regarding the rabbit strain, most studies (62.5%) used New Zealand White rabbits, 18.7% used V-line rabbits, 12.5% used V-line rabbit crosses, and 6.3% of the studies used Rex rabbits.

### 3.2. Growth Performance

Table 2 shows that average daily gain (ADG) and hot carcass yield (HCY) increased in response to BP supplementation (*p* < 0.001). In contrast, a lower feed conversion ratio (FCR) and daily feed intake (DFI) were observed in rabbits supplemented with BP (*p* < 0.001). On the other hand, Table 3 shows that PRO supplementation increased ADG (*p* < 0.001) and HCY (*p* = 0.005); however, FCR and DFI decreased (*p* < 0.001).

### 3.3. Serum Metabolites

Table 4 shows that BP supplementation reduced (*p* < 0.05) the serum concentration of urea, creatinine, cholesterol, total lipids, aspartate aminotransferase (AST), alanine aminotransferase (ALA), and malondialdehyde (MDA). In contrast, higher (*p* < 0.05) serum concentrations of glucose, albumin, globulin, total protein, and higher total antioxidant capacity (TAC) were observed in response to BP supplementation. On the other hand, Table 5 shows that PRO supplementation did not affect (*p* > 0.05) the serum concentration of urea, creatinine, and glucose. However, lower (*p* < 0.05) serum concentrations of cholesterol, total lipids, AST, ALA, and MDA were observed in response to PRO supplementation. In contrast, PRO supplementation increased (*p* < 0.05) the serum concentration of albumin, globulin, total protein, and TAC (Table 4).

### 3.4. Publication Bias and Meta-Regression

Table 2, Table 3, Table 4 and Table 5 show that the Egger asymmetry regression test was not significant (*p* > 0.05) for any of the evaluated variables, indicating no publication bias. On the other hand, Table 2 and Table 3 show that there was significant heterogeneity (Q) (*p* ≤ 0.10) for ADG, DFI, FCR, and HCY. Similarly, Table 4 and Table 5 show significant Q for the serum concentration of urea, creatinine, glucose, cholesterol, total lipids, albumin, globulin, total protein, AST, ALA, MDA, and TAC. However, meta-regression analyses should only be used to obtain reliable results when the variable of interest was reported in at least ten different studies [38]. Therefore, in the present meta-analysis, the meta-regression was only applied to the variables: ADG and DFI (Table 2), glucose, albumin, globulin, and total protein (Table 4) of rabbits supplemented with BP.

Table 6 shows that ADG and serum glucose concentration had no significant relationship (*p* > 0.05) with any of the covariates used. BP dose explained (*p* = 0.026) 27.46% of the observed heterogeneity for serum albumin concentration. The supplementation period explained (*p* < 0.05) 27.57, 30.01, and 45.80% of the heterogeneity observed for the serum concentration of globulin, albumin, and total protein, respectively. Likewise, the age of rabbits explained (*p* = 0.036) 21.83% of the heterogeneity observed for the serum albumin concentration. BP supplementation method explained (*p* < 0.05) 19.70 and 20.38% of the observed heterogeneity for serum albumin concentration and DMI, respectively (Table 6). The covariates sex and rabbit strain had no significant relationship (*p* > 0.05) with ADG, DFI, glucose, albumin, globulin, or total protein.

### 3.5. Subgroup Analysis

**Figure 1:**
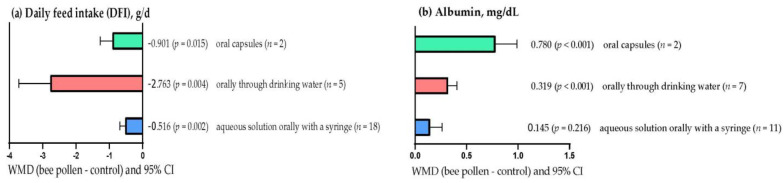
Subgroup analysis (subgroup = supplementation method) of the effect of bee pollen supplementation in rabbits, WMD = weighted mean differences between bee pollen treatments and control.

Figure 1a shows that DFI decreased (*p* < 0.05), regardless of how BP was supplemented. However, the effect was greater (WMD = –2.763 g/d; *p* = 0.004) when BP was administered orally via drinking water than when BP was supplemented via oral capsules (WMD = – 0.901 g/d; *p* = 0.015) or with BP in aqueous solution using an oral syringe (WMD = –0.516 g/d; *p* = 0.002). In contrast, a higher (*p* < 0.001) serum albumin concentration was observed in rabbits when BP was administered orally via drinking water (WMD = 0.319 mg/dL) and via capsules (WMD = 0.780 mg/dL; Figure 1b). However, serum albumin concentration was not affected with BP in aqueous solution using an oral syringe (WMD = 0.145 mg/dL; *p* = 0.216).

**Figure 2:**
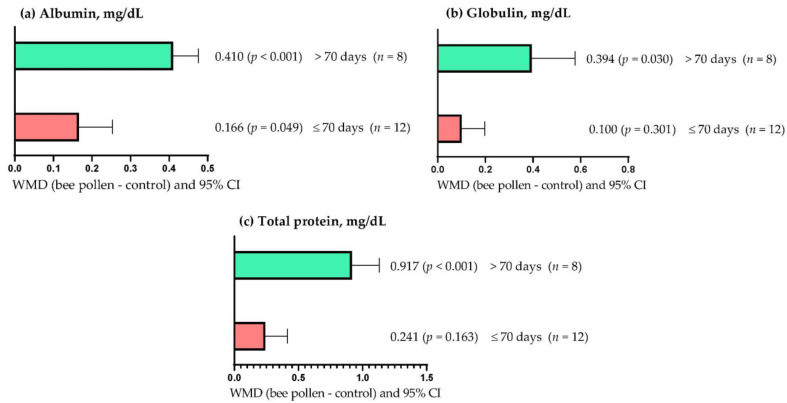
Subgroup analysis (subgroup = supplementation period (days)) of the effect of bee pollen supplementation in rabbits, WMD = weighted mean differences between bee pollen treatments and control.

Figure 2a shows that the serum albumin concentration increased (*p* < 0.05), regardless of the supplementation period used; however, the effect was greater (WMD = 0.410 mg/dL) when BP was supplemented for more than 70 days than periods of up to 70 days (WMD = 0.166 mg/dL). On the other hand, the serum globulin concentration increased (WMD = 0.394 mg/dL; *p* = 0.030) when BP supplementation was longer than 70 days (Figure 2b). However, serum globulin concentration was not affected (WMD = 0.100 mg/dL; *p* = 0.301) when BP supplementation lasted up to 70 days. Figure 2c shows that serum total protein concentration increased when rabbits were supplemented with BP for more than 70 days (WMD = 0.917 mg/dL; *p* < 0.001). However, BP supplementation for up to 70 days did not affect serum total protein concentration (WMD = 0.241 mg/dL; *p* = 0.163).

**Figure 3:**
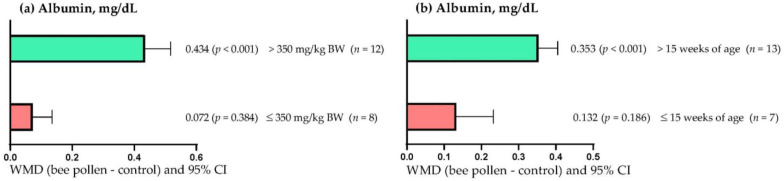
Subgroup analysis (subgroups = bee pollen dose (mg/kg of BW), and age of rabbits (weeks)) of the effect of bee pollen supplementation in rabbits, WMD = weighted mean differences between bee pollen treatments and control.

Figure 3a shows that serum albumin concentration increased when BP doses greater than 350 mg/kg BW were used (WMD = 0.434 mg/dL; *p* < 0.001). However, low doses (≤ 350 mg/kg DM) of BP did not affect serum albumin concentration (WMD = 0.072 mg/dL; *p* = 0.384). On the other hand, Figure 3b shows that the serum albumin concentration increased when BP was administered to rabbits older than 15 weeks of age (WMD = 0.434 mg/dL; *p* < 0.001). However, in rabbits up to 15 weeks of age, BP supplementation did not affect serum albumin concentration (WMD = 0.132 mg/dL; *p* = 0.186).

## 4. Discussion

### 4.1. Growth Performance

Some previously published review articles [6,39] have mentioned that dietary inclusion of BP or PRO could improve the taste of livestock foods. In addition, BP and PRO contain several bioactive compounds (e.g., flavonoids and phenolic acids) with antimicrobial and antioxidant properties [40,41], which could improve feed quality and palatability and lead to higher DFI. However, in the present meta-analysis, lower DFI was observed in response to BP and PRO supplementation. Similar to our results, a meta-analysis conducted by Sadarman et al. [9] reported that PRO supplementation decreased DFI in broilers. The mechanism of action of BP, PRO, and their bioactive metabolites on DFI regulation has not been studied in rabbits. However, recent studies [42,43] have shown that supplementation with FLAs (one of the primary bioactive metabolites of BP and PRO) increases gene expression of bitter taste receptors (TAS2R) in the epithelium of the bovine digestive tract. Activation of TAS2R receptors triggers the release of some anorexigenic molecules (cholecystokinin and peptide YY) [44,45]. Therefore, similar effects of the consumption of BP, PRO, and their flavonoids in the present study partially explain the reduction observed for DFI. On the other hand, BP and PRO contain water-soluble vitamins and minerals [40,41], which according to Attia et al. [14], accelerate nutrient metabolism in rabbits and increase metabolic energy availability. This effect results in lower DFI because in rabbits, as energy availability increases, DFI decreases [46].

In growing rabbits, supplementation with moderate BP doses (500 mg/kg BW) increases the cecal concentration of volatile fatty acids by up to 22% [15]. This effect could result in increased metabolic energy availability and lead to increased ADG since volatile fatty acids provide about 40% of the energy required for maintenance in rabbits [47]. On the other hand, Abdel-Hamid et al. [48] detected increased serum insulin-like growth factor-1 (IGF-1) concentration in rabbits supplemented with BP (250 mg/kg BW). This effect could result in increased ADG since IGF-1 serum levels have been positively correlated with ADG in rabbits [49]. In the present study, BP and PRO supplementation reduced MDA and increased TAC in blood serum. Al-Homidan et al. [18] observed a 21% higher serum concentration of total immunoglobulins (IgM + IgY) in rabbits supplemented with low doses of PRO (250 mg/kg DM). Likewise, it has been reported that supplementation with BP and PRO decreases between 30 and 100% the cecal bacterial count of *Escherichia coli* and *Salmonella spp*. in rabbits [15,18]. These effects could result in better health status of the rabbits and lead to higher ADG. Moreover, in rabbits, flavonoid supplementation increases the serum concentration of growth hormone [50] and the relative cecal abundance of bacterial families (*Peptococcaceae*, *Eubacteriaceae*, and *Syntrophomonadaceae*) that have a positive correlation with weight gain [51]. Similar effects of the consumption of BP, PRO, and their flavonoids in the present meta-analysis would explain the increases observed for ADG.

In rabbits, BP supplementation increases the activity of digestive enzymes (protease, amylase, and lipase) in the intestinal contents and the digestibility of crude fiber, crude protein, and ether extract [15]. Likewise, Waly et al. [16] reported increased digestibility of crude protein and organic matter in rabbits supplemented with low doses (200 mg/kg DM) of PRO. On the other hand, it has been documented that supplementation with BP or PRO increases between 39 and 90% the length of intestinal villi in rabbits [15], which could result in increased nutrient absorption. Additionally, in growing rabbits, North et al. [51] reported that dietary supplementation with flavonoids increases the relative abundance of cecal bacteria (*Clostridiaceae*, *Haloplasmataceae*, and *Erysipelotrichaceae*), which have a negative correlation (r between -0.61 and -0.68) with FCR in rabbits. Similar effects of the consumption of BP, PRO, and their flavonoids in the present meta-analysis partially explain the observed reduction in FCR.

Most studies used New Zealand White rabbits in the present meta-analysis. Therefore, the positive effects of BP and PRO on ADG and FCR should be carefully interpreted, as they may only occur in New Zealand White rabbits. In addition, although the mixture of BP and PRO was not evaluated in this meta-analysis, this combination could act synergistically since the effect of the high flavonoid content of PRO could be potentiated by the high levels of vitamins and minerals provided by BP. Consequently, combining BP and PRO could have a greater positive impact on animal health and performance in rabbits than the individual use of BP or PRO.

### 4.2. Serum Metabolites

According to Hokamp and Nabity [52], serum urea and creatinine concentrations can be used as biomarkers of renal function. For example, high serum urea and creatinine levels indicate loss of nephron function and renal failure [53]. In the present meta-analysis, BP supplementation decreased serum urea and creatinine levels. However, serum urea and creatinine levels in rabbits supplemented with BP or PRO were within the normal ranges (urea: 20–45 mg/dL; creatinine: 0.5–2.5 mg/dL) reported in the literature for healthy rabbits [54]. These results suggest that BP and PRO do not affect the renal health of rabbits. Furthermore, in rabbits, deficiency of any essential amino acid increases catabolism of the remaining amino acids, increases hepatic urea production, and leads to higher serum urea levels [55]. BP contains essential amino acids (methionine, lysine, and threonine, among others) that improve the amino acid balance of rabbits [40], which would explain the lower serum urea concentration observed in response to BP supplementation.

Rabbits supplemented with BP or PRO had serum glucose levels within the normal range (75–155 mg/dL) [54]; however, serum cholesterol concentrations in rabbits supplemented with BP or PRO were above the normal range (10-80 mg/dL) reported in the literature for healthy rabbits [54]. Khalifa et al. [40] mention that BP contains about 30% carbohydrates, mainly glucose and fructose, which partially explains the increase in serum glucose observed in rabbits supplemented with BP. On the other hand, BP and PRO have a wide variety of flavonoids [56,57]. According to Zeka [58], flavonoids can decrease serum cholesterol concentration because they increase the expression of low-density lipoprotein receptors, decrease intestinal cholesterol absorption, and inhibit hepatic cholesterol synthesis. Consequently, the lower serum cholesterol concentration observed in rabbits supplemented with BP and PRO could be related to the flavonoid content of these two products. In addition, BP contains polyunsaturated fatty acids [40], which reduce serum cholesterol levels by inducing the expression of the enzyme cholesterol 7-hydroxylase and increasing receptors for low-density lipoproteins [59].

Serum albumin, globulin, and total protein concentrations in rabbits supplemented with BP or PRO were within normal ranges (albumin: 2.7–5.0 mg/dL; globulin: 1.5–2.7 mg/dL; total protein: 5.4–7.5 mg/dL) reported in the literature for healthy rabbits [54]. In the present study, the higher serum total protein concentration observed in response to BP and PRO supplementation could be related to increased serum albumin and globulin levels. Serum albumin levels are decreased in animals with internal parasitism and when hepatic protein synthesis is low [60]. The present meta-analysis showed a higher serum albumin concentration in response to BP and PRO supplementation. This effect could be related to flavonoids in BP and PRO since flavonoids increase hepatic protein synthesis [61] and decrease internal parasites in rabbits [62]. In addition, BP contains approximately 23% protein [40], which could be related to the higher serum total protein, albumin, and globulin concentrations in BP-supplemented rabbits.

Serum concentrations of aminotransferases such as AST and ALT are used as indicators of hepatocellular damage [63]. For example, AST and ALT levels increase in response to almost all liver diseases, such as fatty liver, cirrhosis, hepatic necrosis, and hepatitis [64]. The present meta-analysis showed lower serum AST and ALT concentrations in response to BP and PRO supplementation. However, serum AST and ALT concentrations in rabbits supplemented with BP or PRO were within the normal ranges (AST: 10–78 UI/dL; ALT: 27.4–72.2 UI/dL) reported in the literature for healthy rabbits [65]. These results indicate that BP and PRO do not affect the liver health of rabbits. 

According to Ghiselli [66], TAC is an integrated parameter that considers the cumulative action of all blood serum antioxidants. Moreover, MDA is frequently used as an indicator of lipid peroxidation [67]. In the present meta-analysis, higher TAC and lower MDA were observed in response to BP and PRO supplementation, suggesting that BP and PRO intake decreases lipid peroxidation and improves total antioxidant status in rabbits. Although little information exists on the antioxidant mechanisms of BP and PRO in rabbits, it has been reported that BP and PRO contain polyphenols (flavonoids and phenolic acids) that are absorbed in the intestinal tract of rodents [56]. Subsequently, these polyphenols can be transferred to the bloodstream, acting directly as exogenous antioxidants and activating transcription factors that increase serum levels of antioxidant enzymes (e.g., catalase) [57,68]. Similar effects of the consumption of BP, PRO, and their polyphenols in the present meta-analysis would explain the increase and reduction observed for TAC and MDA, respectively.

## 5. Conclusions

The present meta-analysis results indicate that bee pollen and propolis reduce feed consumption. Likewise, the results of the subgroup analysis indicated that, for bee pollen, the greatest reduction in feed consumption is obtained when this product is supplemented orally through drinking water. However, bee pollen and propolis can be used as natural growth promoters in rabbits since they increase weight gain and, at the same time, reduce the feed conversion ratio. In addition, bee pollen and propolis supplementation improve antioxidant status in rabbit blood serum. 

## Figures and Tables

**Table 1:** Descriptive statistics of the complete data set for the effect of BP supplementation on rabbits’ diets.

Parameter		Mean		Median		Minimum		Maximum		SD	
Dietary features	NC	Control	BP	Control	BP	Control	BP	Control	BP	Control	BP
DM, g/kg DM	9	901.0	901.1	903.2	903.2	874.7	874.7	917.1	917.1	11.81	11.81
CP, g/kg DM	27	177.8	177.8	180.0	180.0	170.0	170.0	184.0	184.0	5.49	5.49
EE, g/kg DM	12	29.93	29.93	27.75	27.75	26.20	26.20	51.40	51.40	6.89	6.89
NDF, g/kg DM	5	324.1	324.1	326.9	326.9	316.4	316.4	331.1	331.1	7.21	7.21
ADF, g/kg DM	5	154.2	154.2	149.2	149.2	148.1	148.1	163.4	163.4	7.36	7.36
CF, g/kg DM	24	131.8	131.8	130.0	130.0	126.0	126.0	150.0	150.0	6.39	6.39
Ash, g/kg DM	7	94.43	94.43	95.20	95.20	74.8	74.8	103.6	103.6	10.30	10.30
Ca, g/kg DM	3	0.95	0.95	0.87	0.87	0.87	0.87	1.11	1.11	0.13	0.13
P, g/kg DM	3	0.53	0.53	0.41	0.41	0.41	0.41	0.77	0.77	0.20	0.20
DE, MJ/kg DM	26	10.60	10.60	10.47	10.47	9.2	9.2	12.15	12.15	0.69	0.69
BP, mg/kg BW	27	-	374.0	-	335.0	-	100	-	1000	-	195.9
Duration, days		85.0		70.0		28.0		140.0		37.68	

NC = number of comparisons; BP = bee pollen; SD = standard deviation; DM = dry matter; CP = crude protein; EE = ether extract; NDF = neutral detergent fiber; ADF = acid detergent fiber; CF = crude fiber; Ca = calcium; P = phosphorus; DE: digestible energy. In the same column, means followed by different letters differ significantly by the Tukey test (*p* ≤ 0.05).

**Table 2:** Growth performance of rabbits supplemented with bee pollen.

Item	N (NC)					Heterogeneity		Egger Test ^1^
		Control means (SD)	WMD (95 % CI)		*p*-Value	*p*-Value	I^2^ (%)	*p*-Value
ADG, g/d	11 (27)	21.38 (7.33)	1.309 (0.802; 1.816)		<0.001	<0.001	87.91	0.182
DFI, g/d	10 (25)	149.4 (62.9)	−0.935 (−1.343; −0.527)		<0.001	<0.001	81.96	0.061
FCR, DMI/ADG	8 (19)	4.49 (1.00)	−0.708 (−1.021; −0.395)		<0.001	<0.001	99.45	0.353
HCY, %	4 (7)	53.68 (4.53)	2.723 (1.155; 4.290)		<0.001	<0.001	93.10	NA

N: number of studies; NC: number of comparisons; SD: standard deviation; WMD: weighted mean differences between control and treatments supplemented with bee pollen; CI: confidence interval of SMD; p-Value for the χ^2^ test of heterogeneity; I^2^: proportion of total variation of size effect estimates that is due to heterogeneity; ^1^: Egger’s regression asymmetry test; ADG: average daily gain; DFI: daily feed intake; FCR: feed conversion ratio; HCY: hot carcass yield.

**Table 3:** Growth performance of rabbits supplemented with propolis.

Item	N (NC)					Heterogeneity		Egger Test ^1^
		Control means (SD)	WMD (95 % CI)		*p*-Value	*p*-Value	I^2^ (%)	*p*-Value
ADG, g/d	8 (15)	29.53 (6.06)	1.035 (0.441; 1.628)		<0.001	<0.001	76.27	0.117
DFI, g/d	8 (15)	109.44 (26.25)	−0.427 (−0.837; −0.018)		0.041	0.004	55.94	0.196
FCR, DMI/ADG	7 (14)	3.15 (0.87)	−0.442 (−0.560; −0.324)		<0.001	<0.001	81.03	0.095
HCY, %	5 (8)	55.55 (5.63)	3.504 (1.052; 5.957)		0.005	<0.001	90.63	NA

N: number of studies; NC: number of comparisons; SD: standard deviation; WMD: weighted mean differences between control and treatments supplemented with propolis; CI: confidence interval of SMD; *p*-value for the χ^2^ test of heterogeneity; I^2^: proportion of total variation of size effect estimates that is due to heterogeneity; ^1^: Egger’s regression asymmetry test; ADG: average daily gain; DFI: daily feed intake; FCR: feed conversion ratio; HCY: hot carcass yield.

**Table 4:** Serum metabolites of rabbits supplemented with bee pollen.

Item	N (NC)				Heterogeneity		Egger Test ^1^
		Control means (SD)	WMD (95 % CI)	*p*-Value	*p*-Value	I^2^ (%)	*p*-Value
Urea, mg/dL	9 (20)	31.38 (8.94)	−4.023 (−6.827; −1.219)	0.005	<0.001	99.64	0.266
Creatinine, mg/dL	9 (18)	1.17 (0.33)	−0.152 (−0.303; −0.001)	0.049	<0.001	98.95	0.968
Glucose, mg/dL	10 (20)	88.28 (19.24)	13.759 (7.641; 19.876)	<0.001	<0.001	99.69	0.121
Cholesterol, mg/dL	9 (18)	118.80 (56.1)	−11.607 (−13.347; −9.868)	<0.001	<0.001	99.95	0.063
Albumin, mg/dL	10 (20)	3.08 (0.46)	0.268 (0.138; 0.397)	<0.001	<0.001	97.68	0.480
Globulin, mg/dL	10 (20)	2.79 (0.67)	0.196 (0.039; 0.353)	0.015	<0.001	94.78	0.728
Total protein, mg/dL	10 (20)	5.87 (0.92)	0.490 (0.238; 0.742)	<0.001	<0.001	96.58	0.567
AST, UI/dL	8 (17)	50.88 (12.26)	−6.074 (−8.068; −4.080)	<0.001	<0.001	84.99	0.083
ALT, UI/dL	7 (16)	61.91 (13.29)	−6.429 (−8.505; −4.353)	<0.001	<0.001	90.94	0.460
TAC, mmol/L	4 (7)	3.88 (1.22)	0.716 (0.273; 1.159)	0.002	<0.001	99.74	NA
MDA, nmol/mL	3 (6)	6.33 (3.70)	−0.774 (−1.368; −0.180)	0.011	<0.001	89.17	NA

N: number of studies; NC: number of comparisons; SD: standard deviation; WMD: weighted mean differences between control and treatments supplemented with bee pollen; CI: confidence interval of WMD; p-value for the χ^2^ test of heterogeneity; I^2^: proportion of total variation of size effect estimates that is due to heterogeneity; ^1^: Egger’s regression asymmetry test; NA: variables with n < 10 observations, the test does not apply; AST: aspartate aminotransferase; ALT: alanine aminotransferase; MDA: malondialdehyde; TAC: total antioxidant capacity.

**Table 5:** Serum metabolites of rabbits supplemented with propolis.

Item	N (NC)					Heterogeneity		Egger Test ^1^
		Control means (SD)	WMD (95 % CI)		*p*-Value	*p*-Value	I^2^ (%)	*p*-Value
Urea, mg/dL	3 (5)	30.20 (7.59)	−0.842 (−13.942; 12.259)		0.900	<0.001	99.89	NA
Creatinine, mg/dL	3 (5)	0.88 (0.28)	0.151 (−0.121; 0.424)		0.277	<0.001	92.66	NA
Glucose, mg/dL	5 (7)	95.07 (24.56)	7.905 (−5.451; 21.262)		0.246	<0.001	98.16	NA
Cholesterol, mg/dL	5 (7)	107.60 (39.6)	−8.012 (−14.000; −2.024)		0.009	0.030	59.51	NA
Albumin, mg/dL	6 (9)	3.15 (0.52)	0.202 (0.001; 0.404)		0.049	<0.001	82.99	NA
Globulin, mg/dL	6 (9)	2.63 (0.86)	0.275 (0.077; 0.473)		0.006	<0.001	72.13	NA
Total protein, mg/dL	6 (9)	5.78 (1.05)	0.419 (0.072; 0.766)		0.018	<0.001	88.62	NA
AST, UI/dL	5 (7)	50.31 (16.13)	−5.539 (−9.246; 1.543)		0.006	<0.001	81.39	NA
ALT, UI/dL	4 (6)	57.32 (17.71)	−5.571 (−10.333; −0.810)		0.022	<0.001	93.32	NA
TAC, mmol/L	5 (8)	2.47 (1.62)	0.210 (0.036; 0.385)		0.018	<0.001	90.74	NA
MDA, nmol/mL	4 (7)	1.62 (0.58)	−0.213 (−0.294; −0.132)		<0.001	0.045	55.4	NA

N: number of studies; NC: number of comparisons; SD: standard deviation; WMD: weighted mean differences between control and treatments supplemented with propolis; CI: confidence interval of WMD; p-value for the χ^2^ test of heterogeneity; I^2^: proportion of total variation of size effect estimates that is due to heterogeneity; ^1^: Egger’s regression asymmetry test; NA: variables with n < 10 observations, the test does not apply; AST: aspartate aminotransferase; ALT: alanine aminotransferase; MDA: malondialdehyde; TAC: total antioxidant capacity.

**Table 6:** Meta-regression comparing the associations between covariates and measured outcomes.

Parameter	Covariates		QM	Df	*p*-Value	R^2^ (%)
Average daily gain (ADG)	Bee pollen dose		0.351	1	0.554	0.0
	Supplementation period		0.259	1	0.611	0.0
	Rabbit´s age		1.287	1	0.257	0.0
	Supplementation method		2.919	2	0.232	0.0
	Sex		4.093	3	0.252	0.0
	Rabbit strain		1.327	2	0.515	0.0
Daily feed intake (DFI)	Bee pollen dose		0.419	1	0.517	0.0
	Supplementation period		0.005	1	0.942	0.0
	Rabbit´s age		0.136	1	0.713	0.0
	Supplementation method		7.729	2	0.021	20.38
	Sex		6.102	3	0.107	0.0
	Rabbit strain		4.295	2	0.117	5.27
Glucose	Bee pollen dose		0.001	1	0.975	0.0
	Supplementation period		0.502	1	0.479	0.0
	Rabbit´s age		0.036	1	0.850	0.0
	Supplementation method		1.961	2	0.375	0.86
	Sex		2.824	2	0.244	6.72
	Rabbit strain		0.147	2	0.929	2.40
Albumin	Bee pollen dose		4.971	1	0.026	27.46
	Supplementation period		8.465	1	0.004	30.01
	Rabbit´s age		13.467	1	0.036	21.83
	Supplementation method		7.471	2	0.024	19.70
	Sex		7.205	2	0.127	0.0
	Rabbit strain		0.538	2	0.764	0.0
Globulin	Bee pollen dose		0.420	1	0.517	0.0
	Supplementation period		5.680	1	0.017	27.57
	Rabbit´s age		0.008	1	0.928	0.0
	Supplementation method		3.086	2	0.214	7.46
	Sex		6.056	2	0.114	2.18
	Rabbit strain		0.150	2	0.928	0.0
Total protein	Bee pollen dose		2.345	1	0.126	5.49
	Supplementation period		10.458	1	0.001	45.80
	Rabbit´s age		0.003	1	0.960	0.0
	Supplementation method		5.909	2	0.062	10.74
	Sex		6.269	2	0.061	5.76
	Rabbit strain		0.291	2	0.865	0.0

QM: coefficient of moderators; QM is considered significant at *p ≤* 0.05; df: degree of freedom; R^2^: the amount of heterogeneity in the meta-analysis.

## Data Availability

The datasets used and analyzed during the current study are available from the corresponding author upon reasonable request.

## Appendix A

**Table A1:** Summary of the studies included in the meta-analysis.

Author	Country	Product	Duration, d	Age ^1^	Method of supplementation	Dose, mg/kg BW
Abdel-Hamid et al. [48]	Egypt	BP	28	≤15	Aqueous solution orally with a syringe	268, 321
Al-Homidan et al. [18]	Egypt	PRO	42	≤15	Mixed with a basal diet	250, 500
Attia et al. [69]	Egypt	BP	70	>15	Aqueous solution orally with a syringe	54, 120, 171, 309, 600, 904
Attia et al. [70]	Egypt	BP	140	>15	Aqueous solution orally with a syringe	52, 114, 156, 335, 674, 1002
Attia et al. [2]	Egypt	BP, PRO	56	≤15	Aqueous solution orally with a syringe	100, 93
Attia et al. [14]	Egypt	BP, PRO	140	>15	Aqueous solution orally with a syringe	737, 735
Attia et al. [20]	Egypt	BP, PRO	280	>15	Oral capsules	423, 846, 423, 846
Dias et al. [71]	Brazil	BP	82	≤15	Aqueous solution orally with a syringe	1000
El-Hammady et al. [72]	Egypt	BP	56	>15	Orally through drinking water	500, 1000
Hashem et al. [73]	Egypt	PRO	70	>15	Mixed with a basal diet	30
Hashem et al. [1]	Egypt	PRO	35	≤15	Mixed with a basal diet	30, 60
Hassan et al. [17]	Egypt	BP	84	>15	Orally through drinking water	636, 1280
Piza et al. [74]	Brazil	PRO	32	≤15	Mixed with a basal diet	47, 93, 139
Sierra-Galicia et al. [19]	Mexico	BP, PRO	42	≤15	Orally through drinking water	500, 50
Waly et al. [16]	Egypt	PRO	56	≤15	Mixed with a basal diet	100, 150, 200
Zeedan et al. [15]	Egypt	BP	70	≤15	Orally through drinking water	140, 348, 487

BW: body weight; d: days; ^1^: age in weeks; BP: bee pollen; PRO: propolis.

**Table A2:** Descriptive statistics of the complete data set for the effect of PRO supplementation to rabbits’ diets.

Parameter		Mean		Median		Minimum		Maximum		SD	
Dietary features	NC	Control	PRO	Control	PRO	Control	PRO	Control	PRO	Control	PRO
DM, g/kg DM	11	888.4	888.4	878.0	878.0	874.7	874.7	917.1	917.1	16.66	16.66
CP, g/kg DM	14	171.2	171.6	172.8	172.8	160.0	160.0	185.0	185.0	7.91	7.76
EE, g/kg DM	10	44.97	44.97	28.80	28.80	26.20	26.60	78.00	78.00	7.59	7.59
NDF, g/kg DM	8	320.3	320.3	316.4	316.4	314.2	314.2	331.1	331.1	7.56	7.56
ADF, g/kg DM	8	171.8	171.8	162.2	162.2	148.1	148.1	201.2	201.2	24.97	24.97
CF, g/kg DM	12	133.1	133.1	133.5	133.5	126.7	126.7	138.5	138.5	4.24	4.24
Ash, g/kg DM	5	94.12	94.12	100.7	100.7	74.8	74.8	103.6	103.6	12.60	12.60
Ca, g/kg DM	6	6.25	6.25	6.30	6.30	5.90	5.90	6.60	6.60	0.39	0.39
P, g/kg DM	6	3.78	3.78	3.75	3.75	3.50	3.50	4.10	4.10	0.31	0.31
DE, MJ/kg DM	13	11.00	11.00	11.22	11.22	9.40	9.40	11.22	11.22	0.51	0.51
PRO, mg/kg BW	15	-	248	-	139	-	30	-	846	-	259.7
Duration, days		50		42		32		140.0		27.54	

NC = number of comparisons; PRO = propolis; SD = standard deviation; DM = dry matter; CP = crude protein; EE = ether extract; NDF = neutral detergent fiber; ADF = acid detergent fiber; CF = crude fiber; Ca = calcium; P = phosphorus; DE: digestible energy. In the same column, means followed by different letters differ significantly by the Tukey test (*p* ≤ 0.05).
